# Gaps in patient-reported outcome measures integration into the management of lower urinary tract symptoms attributed to benign prostatic hyperplasia (LUTS/BPH): qualitative insights for implementation strategies

**DOI:** 10.1186/s41687-026-01114-0

**Published:** 2026-06-12

**Authors:** Rowida Mohamed, Grace Bardwick, Padraig Carolan, Dacey Maglaque, Pooja Talaty, Francesca R. Farina, Abigail R. Smith, Alexander P. Glaser, James W. Griffith

**Affiliations:** 1 Department of Obstetrics and Gynecology, Pritzker School of Medicine, University of Chicago, Chicago, USA; 2 Division of Biostatistics and Informatics, Department of Preventive Medicine, Feinberg School of Medicine, Northwestern University, Evanston, USA; 3 Division of Urology, Department of Surgery, Endeavor Health, Chicago, USA; 4 Division of Urology, Department of Surgery, Pritzker School of Medicine, University of Chicago, Chicago, USA

**Keywords:** Benign prostatic hyperplasia, Lower urinary tract symptoms, Patient-centered care, Patient‐reported outcome measures, Implementation recommendation

## Abstract

**Background:**

Patient-reported outcome measures (PROMs) are commonly used to monitor lower urinary tract symptoms attributed to benign prostatic hyperplasia (LUTS/BPH). However, there is limited evidence on patients’ perspectives on integrating PROMs into clinical practice and on best practices to improve their implementation to better meet patient needs. The aim of this study is to explore patients’ experiences completing PROMs during routine BPH management, and to develop patient-informed strategies for PROM implementation in urologic care.

**Methodology:**

Virtual semi-structured telephone interviews were conducted with English-speaking patients aged 50 years or older with BPH who attended outpatient urology clinics between May and October 2025. Patients were purposively sampled based on the type of treatment received (medical or surgical). Interview data were analyzed using interpretive description. Themes were synthesized inductively, and practical implementation strategies were developed from participants’ suggestions.

**Results:**

Twenty patients participated (12 received medical treatment and 8 received surgical treatment). Four themes emerged regarding patient perspectives on PROM use in routine care: (1) Perceived benefits—patients viewed PROMs as valuable tools for tracking symptom progression, prompting self-reflection, and facilitating communication among the clinical care team; (2) Questionnaire content—patients questioned the relevance of specific domains, particularly the non-specificity of the PROMIS pain scales as well as mental health, and sexual health items; (3) Perceived barriers—participants criticized the excessive volume of questionnaires; and (4) Considerations for PROM Integration—participants emphasized the importance of visible follow-up actions based on PROM results. Practical recommendations for PROM integration in routine clinical practice included: (1) enhancing patient understanding of PROMs by providing a brief rationale prior to survey administration; (2) implementing a patient-preference PROM workflow in the electronic health record (EHR); and (3) providing tangible actions based on PROM results, such as summarizing scores in plain language and incorporating results into discussions during visits.

**Conclusions:**

This study provides patient-informed insights into the implementation of a broad PROM battery in LUTS/BPH care. Participants emphasized that PROMs are more acceptable when their purpose is clearly explained and when responses are visibly discussed or acted upon in care. Future research should examine how these patient-informed strategies can be operationalized within clinical workflows and integrated with clinician, administrator, and EHR perspectives to support sustainable PROM use in routine practice.

**Supplementary Information:**

The online version contains supplementary material available at 10.1186/s41687-026-01114-0.

## Background

Benign prostatic hyperplasia (BPH) is a common condition among older men that often results in lower urinary tract symptoms (LUTS) such as urgency, frequency, nocturia, and incomplete emptying [1]. The American Urological Association Symptom Index (AUA-SI) has long served as the standard patient-reported outcome measure (PROM) for assessing LUTS. However, the AUA-SI does not fully capture other important symptom domains, including urinary pain and incontinence [2]. To address these limitations, the Lower Urinary Tract Dysfunction Research Network (LURN) developed the Symptom Index-29 (SI-29) and its shorter form, the SI-10 [3, 4]. Integrating such PROMs into routine clinical practice has the potential to support shared decision-making, strengthen patient–clinician communication, and enhance patient-centered care, ultimately improving patient outcomes and quality of life [5]. Although PROMs are included in both U.S. and European BPH management guidelines for symptom monitoring, their systematic integration into routine clinical workflows remains a significant challenge [6, 7].

Healthcare systems are placing greater emphasis on PROM-based performance metrics as indicators of care quality and patient health outcomes [8]. In parallel, value-based care models, such as the Centers for Medicare and Medicaid Services’ (CMS) proposed Oncology Care First (OCF) model, emphasize the importance of routine symptom monitoring, with symptom monitoring using PROMs as a core requirement [9]. Although no current payment model in urology mandates PROM collection, the broader shift toward value-based care and PROM-driven metrics may accelerate PROM adoption. This trend highlights the need for pragmatic solutions to facilitate the integration of PROMs into routine clinical practice.

Implementation strategies provide actionable solutions to overcome barriers to PROM use, tailored to specific local contexts [10]. When applied effectively, such strategies can improve acceptability, enhance patient engagement, promote adoption, and support the successful and sustainable integration of PROMs into routine clinical practice. Within LUTS/BPH care, previous research from LURN supports assessing symptoms beyond traditional LUTS, including depression, anxiety, sleep disturbance, and physical function, which are associated with male LUTS in both cross-sectional and longitudinal studies, and assist in capturing clinically meaningful aspects of patient phenotypes [11–13]. Building upon this foundation, the present study investigates patients’ experiences with a comprehensive, LURN-informed whole-person assessment battery in clinical practice and identifies patient-informed strategies to facilitate its integration into LUTS/BPH care.

## Methods

### Study design

This study is part of a larger qualitative study aimed at exploring the unmet needs of patients with BPH [14]. The protocol is registered at ClinicalTrials.gov (NCT05898932). Reporting of findings was guided by the Consolidated Criteria for Reporting Qualitative Research (COREQ) (Supplemental Material 1, Table S1) [15].

### Participants, recruitment, and setting

We prospectively recruited patients from the outpatient urology clinics at Endeavor Health —formerly known as NorthShore University HealthSystem (“NorthShore”) — in Chicago, USA. Purposive sampling was used to assemble a clinically diverse cohort based on treatment plan (surgical vs. medical management) [16]. Eligible participants included English-speaking adults aged 50 years or older with a diagnosis of LUTS/BPH who were receiving standard care as determined by their treating urologist. Demographic and clinical characteristics (e.g., age, race, ethnicity, education, comorbidities) were extracted from the electronic health record (EHR). LUTS symptom profiles were assessed using PROMs: AUA-SI and LURN SI-29.

Participants were purposively recruited from the parent study using maximum variation sampling to capture a broad range of experiences with PROM completion across treatment pathways, LUTS severity, and comorbidity burden [17]. A minimum of 20 interviews was established a priori for this qualitative study, with additional interviews planned if thematic saturation was not achieved. Prior empirical work suggests that saturation in qualitative interview studies is often reached within 9 to 17 interviews [18]. Twenty participants were interviewed, including 12 who received medical treatment and 8 who received surgical treatment. These subgroup sizes were selected to ensure adequate representation of each treatment pathway within the overall sample, rather than to support formal between-group comparisons. The final distribution broadly reflected the proportion of participants in the parent cohort who received medical versus surgical treatment during the interview period. The qualitative sample was drawn from a subset of eligible participants during a defined recruitment period. Of [40] participants approached [15], declined participation [5], did not respond or could not be scheduled, and [20] completed interviews. Due to purposive recruitment and constraints imposed by the qualitative study timeline, not all eligible participants were approached. Of those who declined to participate, only two had missing PROM data, while the remainder had complete PROM data comparable to that of interviewed participants. Further details regarding study design, sampling procedures, and data collection are available in the published study protocol [14].

### Procedures

Participants completed a comprehensive PROM battery as part of the parent prospective observational study. The selection of PROMs was guided by previous findings from LURN I and II studies, as well as the broader phenotyping objectives of the parent study. Previous LURN research has demonstrated that LUTS heterogeneity cannot be explained solely by urinary symptoms; additional domains such as bowel symptoms, sleep disturbance, mental health, sexual function, physical function, and bladder diary parameters may also differ across symptom phenotypes or be associated with urinary symptom burden. Therefore, the parent study incorporated core LUTS/ BPH symptom measures (AUA-SI and LURN SI-29) in addition to selected broader domains to facilitate a comprehensive characterization of patients’ symptom experiences. The complete list of administered PROMs is provided in Supplemental Material S2.

A semi-structured qualitative interview guide (Supplemental Material 1, Table S2) was employed to investigate patients’ experiences with completing PROMs and to gather their perspectives on the effective integration of PROMs into routine clinical care. The development of the interview guide was led by a postdoctoral scholar with a PhD in health services and outcomes research, specializing in measurement science and patient-centered research, under the supervision of a professor and clinical psychologist with experience in PROMs and LUTS/BPH research. The guide was subsequently refined with input from a multidisciplinary study team comprising investigators with expertise in LUTS/BPH research, urology, patient-reported outcomes, biostatistics, and qualitative methods. Interview questions were informed by the study objectives and relevant literature regarding PROM use in clinical practice [19–21].

Interviews were conducted by a clinical research coordinator (G.B.), who holds an MPH and prior training in qualitative methods and interviewing. The interviewer was not involved in participants’ clinical care and had no prior relationship with participants beyond study-related contact. The interviewer was blinded to participants’ PROM responses. Virtual, 30-minute semi-structured interviews were conducted between May and October 2025. Except for one interview in which a participant’s spouse was present, no one else was present during the interviews. All interviews were audio-recorded and transcribed verbatim using a HIPAA-compliant transcription service. Participants provided written informed consent and audio-recorded verbal consent before participation. Each participant received a $40 incentive for completing the interview. In the results, the participants’ quotations are presented in italics.

### Analysis

Interview data were analyzed using an interpretive description approach, a qualitative method aimed at providing a detailed understanding of the subject matter. This methodology seeks to generate knowledge applicable to practical contexts [22]. An initial codebook was developed inductively by the first author (R.M.) through review of five transcripts [23]. Two researchers (R.M. and G.B.) discussed and revised the preliminary codebook. The researchers independently applied the updated codebook to sets of 2–3 transcripts each. Following each set, the researchers compared coding, resolved discrepancies by consensus, and iteratively refined existing codes or added new ones as they emerged. Regular meetings with the senior author (JWG.) informed the coding process and resolved any discrepancies. Saturation was defined as the point at which no substantively new themes or insights emerged, confirmed by at least three subsequent interviews. In this study, saturation was assessed retrospectively across the entire sample rather than used prospectively to determine when recruitment should cease. All transcripts were double-coded. Relevant quotations were organized by interview numbers and treatment group (medical vs. surgical).

### Synthesis

Following completion of primary coding, a secondary interpretive synthesis was conducted to examine the practice implications of PROM use in LUTS/BPH care. In alignment with interpretive description methodology, this analytic phase extended beyond topical categorization to analyze patterns and clinically meaningful interpretations across participant accounts. Coded excerpts related to PROM burden, administration preferences, and suggestions for improving PROM use in care were revisited and compared across interviews to identify recurring convergences and areas of divergence. Through iterative team discussions, these categories were further interpreted and synthesized into higher-order implementation priorities, reflecting both participant recommendations and the ways in which their experiences, preferences, and expectations clustered to inform more acceptable and feasible PROM implementation in clinical practice. Demographic characteristics were summarized using descriptive statistics in R (Version 4.3.3), and qualitative data analysis was conducted in Dedoose software (version 10.0.59) [24].

## Results

Twenty patients were interviewed (12 medical, 8 surgical). Demographic and clinical characteristics of the cohort are presented in Table 1. Surgical patients were older than those in the medical management group. Both groups were White, non-Hispanic. Hypertension was more common in surgical patients, while depression and obstructive sleep apnea with Continuous Positive Airway Pressure (CPAP) machine use appeared only in the medical group. Results from PROMs indicated similarities in overall AUA-SI scores but notable differences in specific LURN SI-29 domains. Surgical patients reported greater urgency, incontinence, and slightly more urinary pain.

**Table 1:** Patient demographics

	Medical (*N* = 12)	Surgical (*N* = 8)	Overall (*N* = 20)
**Age (years)**			
Mean (SD)	67.3 (8.21)	75.0 (9.07)	70.4 (9.20)
**Race**,** n (%)**			
Caucasian or White	12 (100%)	8 (100%)	20 (100%)
**Ethnicity**,** n (%)**			
Not Hispanic or Latino	12 (100%)	8 (100%)	20 (100%)
**BMI (kg/m** ^**2**^ **)**			
Mean (SD)	27.4 (3.85)	26.5 (2.21)	27.0 (3.25)
**Education**,** n (%)**			
High school/some college	1 (8.3%)	1 (12.5%)	2 (10.0%)
Bachelor’s degree	6 (50.0%)	4 (50.0%)	10 (50.0%)
Graduate degree	5 (41.7%)	3 (37.5%)	8 (40.0%)
**Comorbidities Extracted from the EHR**			
**Anxiety**,** n (%)**	1 (8.3%)	1 (12.5%)	2 (10.0%)
**Depression**,** n (%)**	2 (16.7%)	0 (0%)	2 (10.0%)
**Diabetes Mellitus**,** n (%)**			
Type I	0 (0%)	0 (0%)	0 (0%)
Type II	1 (8.3%)	1 (12.5%)	2 (10.0%)
**Hypertension**,** n (%)**	7 (58.3%)	7 (87.5%)	14 (70.0%)
**Obstructive Sleep Apnea**,** n (%)**			
Do not use CPAP	7 (58.3%)	2 (25.0%)	9 (45.0%)
Use CPAP	3 (25.0%)	0 (0%)	3 (15.0%)
**Obesity**,** n (%)**	4 (33.3%)	0 (0%)	4 (20.0%)
**Patient-reported outcome measures (PROMs)**,** mean (SD)**			
**AUA-SI**	17.3 (5.43)	17.7 (9.88)	17.5 (7.11)
**AUA-SI QOL**	3.83 (1.03)	3.88 (1.36)	3.85 (1.14)
**LURN SI-29 Total score**	27.1 (7.66)	31.7 (18.7)	28.8 (12.6)
**LURN SI-29 Incontinence**	3.47 (4.29)	10.1 (12.9)	5.92 (8.82)
**LURN SI-29 Pain**	7.29 (8.77)	9.82 (17.6)	8.22 (12.3)
**LURN SI-29 Voiding**	42.5 (19.6)	43.6 (21.4)	42.9 (19.7)
**LURN SI-29 Urgency**	27.1 (18.2)	40.5 (31.0)	32.0 (23.8)
**LURN SI-29 Nocturia**	70.2 (34.7)	69.4 (27.9)	69.9 (31.5)
**LURN SI-29 Bother**	1.08 (0.515)	1.43 (0.976)	1.21 (0.713)

**Abbreviations**: AUA-SI: American Urological Association Symptom Index; LURN SI-29: Lower Urinary Tract Dysfunction Research Network Symptom Index-29; BMI: Body Mass Index; CPAP: Continuous Positive Airway Pressure; EHR: Electronic health record; QoL: quality of life

By interview 16, the core thematic categories were established, and additional interviews were no longer contributing substantively new themes, codes, or implementation-relevant perspectives. The final four interviews primarily reinforced existing themes and added interpretive nuance, contextual variation, and illustrative quotations, rather than introducing new thematic categories. Analysis revealed four overarching themes: (1) Perceived benefits, (2) Questionnaire content, (3) Perceived Barriers, and (4) Practical Considerations for PROM Integration in routine clinical practice. The codebook is available in Supplemental Material 1, Table S3. Each theme is described below with illustrative quotations.

### Theme 1: Perceived benefits

#### Care coordination

Patients emphasized that PROMs could enhance communication and coordination across members of their care team by making the results accessible to all relevant providers. Some preferred having their PROM responses automatically shared with their primary care provider. One participant emphasized that sharing PROM results among members of the health care team could help avoid unnecessary duplication of effort, as well as maintain continuity of care, noting: *“When you go into a doctor’s office*,* the last thing you wanna do is have to sit there and fill out paperwork*,* especially when you’ve done it before. So*,* my point is*,* why not use information that you already have for people and use that over time? Continue to update it*,*” (Interview 15*,* Medical arm).* Notably, while many patients supported broad sharing of PROM results, some emphasized patient choice and control in the process. One participant emphasized the importance of remaining informed alongside their providers: *“They can copy my primary… but I would also want to know about it” (Interview 10*,* Medical arm).* Another stressed that not all patients may want automatic distribution of their PROM results: “*It might be good to get the… patient’s desire to have that kind of feedback. People might not want it… I prefer that [results] be shared back with me so I could… make a decision about them” (Interview 17*,* surgical arm).*

#### Clinically meaningful application

Patients described how PROMs could be applied in a clinically meaningful way in their medical care, both to monitor individual progress and to inform treatment decisions. Several participants emphasized the importance of PROMs in tracking changes in their condition over time. One patient explained, *“Well*,* the only thing that comes to mind is if there’s a way to track progress or differences—because needless to say*,* if I’m seeing a doctor every six months to a year*,* I don’t necessarily think how things were six months to a year ago.” (Interview 14*,* Medical arm).* Patients also emphasized the importance of PROMs in influencing treatment decisions. One of them articulated that, depending on how they answer, clinicians might suggest or preclude procedures: *“It might be helpful if I answer in a way that tells them how they might solve my problem…it might turn out that because of what I’ve answered*,* they don’t feel that I need Aquablation or steam or roto-rooter or whatever other else ways there are…” (Interview 9*,* surgical arm).* Beyond their individual care, some participants envisioned PROMs contributing to improvements for broader patient populations. One explained, *“I’m hoping that it helps treatment down the road*,* either for myself*,* but it was more for just the general prostate or BPH awareness and treatment in the future to have more knowledge and figuring out different paths of treating.” (Interview 10*,* Medical arm)*.

#### Patient engagement

Participants described how completing PROMs prompted them to reflect on their symptoms and behaviors that might otherwise go unnoticed. One participant noted that completing the questionnaires made them “*more aware of scenarios that [they] had not been aware of” (Interview 1*,* Medical arm).* Another explained that responding to these questions encouraged them to *“pay more attention to what [they were] eating and drinking and exercising” (Interview 7*,* Medical arm).* For some, completing the questionnaires motivated them to play a more active role in their care. One participant explained how PROMs prompt them to bring additional questions or concerns to their healthcare team: *“Reading through it*,* if there were some questions that I did have*,* [I’d think]*,* ‘Well*,* maybe I should share that with the appropriate person’” (Interview 4*,* Medical arm).*

### Theme 2: Practical considerations for PROM integration

#### Interpretation / presentation of PROM data

Patients emphasized that PROM results were only meaningful when presented clearly and in an interpretable way. Many wanted concise physician-led explanations that paralleled how providers typically summarize laboratory or physical exam results. As one participant explained: *“I think the next time I see the urologist*,* I would prefer some kind of a quick summary from him of what*,* from his perspective*,* all of this means. Just like when I have a physical with my internist*,* he gives me an update the next time I see him on his impressions.” (Interview 3*,* Surgical arm).* Others desired written reports that not only summarized scores but also highlighted potential risks and contributing factors. One participant suggested, *“Yeah. Just to have a report where you can then kinda look to see where I am in terms of the world and whether or not I’m at risk for something … Definitely a section that highlights anything that I’m at a high risk for…and do any of those items have any sort of play as to why I’m having the condition that I have. So*,* if they’re putting that together*,* and they have science behind what may be effective*,* maybe it is sleep; maybe it is stress—I would love them to be able to highlight that.” (Interview 15*,* Surgical arm)*.

Some participants emphasized that simply seeing their individual responses was not enough; they wanted context and comparisons with other patients with BPH. Visual benchmarks were also considered useful, such as graphs showing how individual responses compared with broader patient populations. One participant described: *“It would be wonderful to see sort of a bar graph of the population and then a line that represents my responses or a dot that would represent my response within the bar graph. That might be… reassuring. I think*,* just to see where you kind of fall…I suppose some component of age might be interesting as well in that mix… It’d be good to know something about other people’s experiences with the surgery in terms of success rates” (Interview 17*,* Surgical arm).*

#### Actionable recommendations

Patients highlighted that the usefulness of PROMs ultimately relies on whether the results lead to tangible clinical actions. Several participants expressed frustration with data collection without follow-up, stressing that PROMs should drive decision-making. One patient explained, *“As long as they take the information and use it…I mean*,* otherwise*,* what’s the worth of doing something like this…Yeah*,* you want to see some action come along with whatever results you get … I would want the medical provider to be aggressive when it comes to my health. I want them to be like*,* ‘Okay*,* let’s do this*,*’ instead of*,* ‘Well*,* let’s see if this works.’ So hopefully*,* they would take that information*,* be aggressive with it*,* and start doing the things that need to be done instead of… just kind of ‘We’ll wait till the next.’” (Interview 12*,* Medical arm).*

Participants also underscored that PROMs should be acted upon in the same way as other clinical data. One drew a direct analogy to laboratory testing, explaining, *“If the results of the questionnaire alter or support actions that the physician is taking or recommending*,* that would be appropriate…if you have a blood test that shows that you have an infection in your body right now*,* you have a careful review to find out where it is…If you learn something*,* you should be able to act upon it or take action on it. In other words*,* why answer the question?” (Interview 3*,* Surgical arm)*.

Other participants expressed similar expectations that PROMs should trigger further evaluation or recommendations when concerning responses arose. As one participant noted, *“I sort of expected the questionnaires to be analyzed by a professional and if there were a topic that sort of jumped out*,* that would have resulted in a recommendation that I get further assistance—that’s what I expected.” (Interview 18*,* Surgical arm).*

#### Whole-person symptom recognition

Patients emphasized the importance of PROMs in capturing symptoms that extended beyond their prostate condition, underscoring the value of a holistic approach to care. Several participants expected that physicians would acknowledge such findings and act on them, rather than overlooking them as outside the scope of their specialty. One explained, *“A physician should not be limited to making comments only about their narrow area of specialization” (Interview 3*,* Surgical arm).* Another participant emphasized that if PROMs revealed additional conditions, they should be addressed directly: *“If the urologist notices something … either side effect or other conditions*,* I would certainly want them to bring it up” (Interview 2*,* Medical arm).*

Participants also highlighted the importance of referrals when PROMs uncovered issues requiring attention from other specialties. As one noted, *“I would expect that*,* if the specialist found something that was not—you know*,* he’d have to redirect that back to my primary” (Interview 12*,* Medical arm).* Another added, *“The physician can tell me that*,* ‘Yeah*,* the sleep apnea could be contributing*,* and you should take care of that and maybe give me recommendations on where to go from there” (Interview 5*,* Medical arm).*

For some, this broader scope was seen as the actual value of PROMs in addressing health more holistically. As one participant explained, *“I would love for them to be able to put two and two together and let me know. Again*,* I think that’s the purpose of all those surveys was to try to look at a more holistic view of my life and things that I’m going through” (Interview 15*,* Medical arm).*

#### Patient education about PROMs

Many participants expressed uncertainty about why specific questions were asked or how their responses related to their condition. Certain items occasionally caused confusion, particularly when they addressed symptoms patients did not readily associate with BPH. For instance, one participant reacted to a question about bowel symptoms, *“I was very surprised to see the diarrhea on there. I’m curious about that because…but when I read that question*,* I was like*,* ‘is there a connection here?” (Interview 17*,* Surgical arm).* Another participant reflected: *“Because I didn’t know how it worked. I mean*,* maybe there is something with the bowel movement*,* with urine is tied together somehow. Uh*,* nobody told me that” (Interview 4*,* Medical arm).*

Some participants questioned the focus on specific domains, such as sexual function, without understanding their relevance: “I didn’t understand why there seemed to be an emphasis on sexual function. What is that? I mean, realizing it’s happening in the same organ but—outside of that, I didn’t understand why there was so much emphasis on that in relation to this particular surgery.” (Interview 8, Surgical arm).

Questions related to mental health were also sometimes perceived as unrelated to participants’ primary concerns. One participant remarked, *“I didn’t need the ones about the mental health… I don’t think they have much to do with my urinary problem”* (*Interview 9*,* Surgical arm*). Similarly, another participant reflected that these items felt somewhat disconnected from their experience, explaining, *“…and maybe mental health seems to be kinda more off the track … why I would connect to the urinary issues.”* (*Interview 15*,* Medical arm*).

Several participants suggested that questionnaires should include an explanation of their purpose. As one explained: “I guess an explanation, … like, ‘This is why we want to do the questionnaire—and here’s the benefits of doing the questionnaire.’ … At the conclusion, you’d like to see why we did this, what this means. Is that fair?” (Interview 11, Surgical arm).

#### Preferred feedback format

Participants expressed diverse preferences for how PROM results should be communicated to them. Many appreciated the convenience of receiving results electronically. As one participant explained: *“Sending it electronically in an email or whatever -- that would seem to be the simplest way of doing it*,* the most efficient way of doing it. Because my assumption is that most of the stuff online could save you some money. You don’t have to print everything and mail it out” (Interview 4*,* Medical arm).* Some patients emphasized the security and convenience of portals: *“I did get an email*,* and then*,* I was able to find a message on [patient portal] … I’m totally comfortable with that. I’d rather not have to kind of get something in the mail and have to send it back or to fax it… It’s just more convenient to do it digitally…I feel like it’s pretty secure with [patient portal].” (Interview 16*,* Medical arm)*.

At the same time, several participants emphasized the value of in-person discussions with their physicians about the PROM results. One explained: *“I always appreciate a discussion…. I’d always much rather talk to somebody live if that’s possible.” (Interview 13*,* surgical arm).* Another reinforced this preference: *“I think—the urologist conversation is absolutely always the best…you don’t have to type; you don’t have to open portals…one thing leads to another conversation.” (Interview 9*,* surgical arm).*

Others suggested that a combination of written and verbal communication was most useful. As one patient put it: “I would say it’d be good to have some kind of written thing, but conversation with a urologist could also be beneficial … I would probably say both those two” (Interview 10, Medical arm). Finally, some participants preferred physical copies, with one noting, “I like hard copies of everything. So, if you’re gonna send something… I’d rather get them in the mail than have to diddle around with them on the screen and then print them up” (Interview 8, surgical arm).

### Theme 3: Questionnaire content

#### Perceptions of questionnaire’s topics (relevant, sensitive topics)

Patients’ appraisals of the relevance of questionnaire domains varied depending on their personal experiences. Many found the urinary symptom items directly relevant and central to their condition. One participant explained, *“I think it was the urinary ones that were the most applicable… because it was closer to my problems I was having” (Interview 14*,* Medical arm).* In contrast, participants were less receptive to domains that did not align with their lived experiences or reasons for seeking urologic care. Pain was a frequent example, with several participants reporting that it was “out of the picture” since they experienced none: *“When it comes to pain*,* I didn’t have any pain. No pain at all. So that was out of the picture as part of the questionnaire” (Interview 6*,* Medical arm).* Also, several patients described items on bowel function as misaligned with their condition: *“I don’t have diarrhea. I don’t have constipation… so all those are off the board” (Interview 18*,* Surgical arm).* For some, questions about sexual health were irrelevant because they were no longer sexually active: *“I’m not having sex anymore. So that was the least relevant for me” (Interview 11*,* Surgical arm).*

Mental and sexual health questions were sometimes perceived as inappropriate or even offensive. One participant commented, “I’m always a little bit surprised and almost insulted that the mental health questionnaire comes up all the time… to me it’s just silliness. It’s almost insulting… If I have a problem, I will seek out a psychologist or psychiatrist. But other than that, thank you, I’m just fine” (Interview 3, Surgical arm). Another patient explained that sexual health is something he felt uncomfortable addressing in a survey context, “I have some erectile dysfunction issues, so I did not answer those two questionnaires. That’s something that Dr. xx and I will discuss after I’ve had the next procedure” (Interview 3, Surgical arm).

Several participants commented on the repetitiveness of the questionnaires, noting that similar questions appeared multiple times across different sections. Another observed that, *“A lot of them seem to ask the same questions*,* maybe in a little bit of a different way*,* but I feel some of them get a little repetitive” (Interview 10*,* Medical arm).*

#### Perceptions of response options

Participants reflected not only on the content of the questionnaires but also on the structure of the response options provided. Some found the fixed-choice categories to be limiting, particularly when their symptoms varied in frequency or intensity. One participant described difficulty answering a question about split stream: *“Well*,* it happens*,* but not all the time. And the question didn’t ask me if it happened all the time or if it had ever happened. So*,* I didn’t know how to answer that” (Interview 18*,* Surgical arm).* Another participant noted that situational health experiences were difficult to answer without frequency anchors, *“Some of the questions I’m answering are more situational. Like*,* do you ever feel this way? Yes*,* maybe I do. But when it’s framed as ‘how many times a day or how many times a week*,*’ that’s a little more beneficial”* (*Interview 10*,* Medical arm*). At the same time, patients pointed out that the lack of space for open-ended input limited their ability to contextualize or clarify their answers. One explained, *“There’s no place to put any comments… I recognize if you’re trying to give us a score or quantify it*,* you have to set categories*,* which can be arbitrary” (Interview 2*,* Medical arm).*

#### Recall periods

Participants frequently commented on the recall time frames used in the questionnaires, particularly the standard “last seven days” framing. They described these recall windows as restrictive and sometimes unrepresentative of their condition. One participant explained, *“Some of the questions*,* you know—the time frame of the last seven days—I think [is] a little restrictive… the condition changes sort of week to week. So*,* you know*,* the last seven days might not be quite the same as the previous seven days” (Interview 2*,* Medical arm).* Others found it cognitively difficult to apply a short recall period when symptoms fluctuated over more extended periods. One participant reflected, *“They’re all very valuable questions*,* but they can be very difficult to answer. You know*,* I mean*,* in the last seven days… on the eighth day… I felt this way. I’d better put down that*,* although it isn’t– it isn’t during the last seven days …it’s sort of a mind game. Instead of thinking about it*,* you just put down your initial reaction and move on”* (*Interview 4*,* Medical arm*).

#### Wording clarity

Participants discussed the challenges they faced when interpreting specific questionnaire items, noting that unclear or ambiguous wording often made it difficult to provide accurate responses. For some, the terminology was confusing or unfamiliar, causing them to question what the items actually meant. For example, one patient noted confusion with the term *“split stream”* and wondered, *“What does the split stream mean?”* (*Interview 7*,* Medical arm*). Another mentioned, *“Split*,* I didn’t quite understand”* (*Interview 9*,* Surgical arm*). Several participants noted that the PROMIS pain questions felt overly broad and did not distinguish between urinary pain and pain arising from other health issues. One participant reflected, *“You ask about pain*,* and… you should ask*,* ‘what kind of pain?’ My pain is not because of the prostate. My pain is because I am getting over spinal fusion… but you don’t differentiate what kind of pain in the questionnaires”* (*Interview 3*,* Surgical arm*). Another participant explained, *“They talked about walking up and downstairs. I mean*,* I got back problems*,* so that’s what I was relating it [pain questions] to. I don’t know if that really was what they were looking for”* (*Interview 7*,* Medical arm*).

#### Perceived comprehensiveness

Several participants described the set of questionnaires administered as inclusive. For example, one participant noted, *“They were certainly very inclusive. They covered everything” (Interview 13*,* Surgical arm).* Despite this overall sense of thoroughness, some participants identified essential gaps in symptom coverage. One participant highlighted insufficient attention to nocturia and morning urinary patterns; although the LURN SI-29 and AUA-SI include questions about nighttime symptoms and a weak urine stream, one participant commented: *“There weren’t enough questions for me about nighttime. You know. I wake up two*,* maybe three times a night to urinate. And then in the morning*,* it’s very… weak stream… The questionnaires didn’t really address that aspect” (Interview 2*,* Medical arm).*

### Theme 4: Perceived barriers

#### Physical burden of voiding diaries

Although this study was focused on PROMs, completing the voiding diary was described as physically disruptive and burdensome. Several participants highlighted the difficulty of tracking nocturia episodes at night, noting how the task itself interfered with their sleep. One described, *“Voiding diaries… it was challenging*,* especially in the middle of the night… it woke me up more than what I would like to be woken up when I’m going to the bathroom … In the middle of the night when I have to record time*,* it wakes me up*,* and then it’s harder for me to go back to sleep… I got up at 3:00 to pee*,* but I had to look at what time it was so I could make a note of that. And by then*,* I was awake*,* so then I was up for an hour ’cause I couldn’t fall back asleep”* (*Interview 1*,* Medical arm*). Another participant shared, *“This void thing was hard for me because I had to… grab the vessel*,* pee into it*,* turn on the light*,* put on my glasses*,* read the amount*,* and then go chart it on MyChart and wash out the container. If I don’t fall asleep fast enough*,* then I’m up. One time… at 3:30… that was it*,* I was up for the rest of the day”* (*Interview 11*,* Surgical arm*).

#### Overwhelming Quantity of Questionnaires

Participants reflected on the volume of questionnaires they were asked to complete. For some, the number felt excessive, with one participant noting, *“It just seemed like a little bit overly comprehensive and that you could boil the eight of them down into four and cover all the topics you needed”* (Interview 3, Surgical arm). To make the process more manageable, participants recommended clear upfront communication, such as an estimated time commitment, with one participant describing, *“When I saw all the questionnaires, I’m like, ‘Holy cow, it’s a lot of questionnaires’...Just having more of a heads up as to what I’m filling out and how long it would take overall would be nice”* (Interview 15, Medical arm).

### Implementaion strategies

The interpretive synthesis showed that participants’ accounts converged around a set of interrelated implementation needs. Across interviews, concerns about the unclear purpose of cetrain questionnaires, the burden associated with survey length or content, limited personalization, and the absence of visible clinical response were closely linked to participants’ preferences for clearer explanations, more flexible administration, and more meaningful discussion or follow-up based on PROM results. Considered together, these patterned accounts pointed to several higher-order implementation priorities: (1) enhancing patient understanding of PROMs through clear explanations of their purpose, relevance, and expected completion time; (2) Embedding a patient-preference workflow into the EHR allows patients to tailor PROM administration and share results according to their preferences; and (3) providing tangible actions based on PROM results by ensuring that results are reviewed, acknowledged, and discussed during visits. These synthesized priorities informed the patient-centered implementation strategies presented in Table 2.

**Table 2:** Implementation strategies for routine collection of patient-reported outcome measures (PROMs)

Strategy	Barrier Addressed	Actionable Component	Supporting Quotations (Participant ID)
1-Enhancing patient understanding of PROMs	Confusion about the purpose and relevance of PROMs; uncertainty about how results are used	a) Provide patients with an upfront estimate of survey time (e.g., “~10 min”) b) Explain in plain language how PROMs will inform care c) Explain in plain language why PROMs include non-urinary questions	“I guess an explanation, … like, ‘This is why we want to do the questionnaire—and here’s the benefits of doing the questionnaire.’ … At the conclusion, you’d like to see why we did this, what this means.” (Interview 11, Surgical arm)
2. Implementing a patient-preference workflow in the EHR	Lack of personalization in how PROMs are administered and shared; patients differ in communication preferences	a) Allow patients to opt out of PROM domains perceived as irrelevant or uncomfortable to complete b) Offer optional open-ended space for patients to elaborate on their answers. c) Offer preferred delivery formats (MyChart upload, one-page printout, mailed copy) d) Include a checkbox to indicate if patients want providers to address non-urologic results (e.g., sleep, depression) e) Enable routing preferences for sharing PROMs with specific providers	“It might be good to get the patient’s desire to have that kind of feedback. People might not want it… I prefer that [results] be shared back with me so I could… make a decision about them.” (Interview 17, Surgical arm)
3. Providing tangible actions based on PROMs results	Patients lose motivation when results are not visibly used or discussed	a) Add MyChart auto-notification once results are reviewed b) Display key PROM trends in visit summaries c) Have the provider acknowledge PROM data in each visit (e.g., “I see your sleep score improved”)	“As long as they take the information and use it… Otherwise, what’s the worth of doing something like this? … You want to see some action come along with whatever results you get.” (Interview 12, Medical arm)

## Discussion

This study explored patients’ perspectives on the gaps, challenges, and opportunities related to integrating PROMs into LUTS/BPH care. Participants recognized the value of PROMs for longitudinal symptom tracking, clinical decision-making, care coordination, and identifying health concerns that may extend beyond urology. However, they emphasized that PROMs are meaningful only when their purpose is clear, and their results are visibly reviewed, discussed, or acted upon. Challenges related to questionnaire burden, unclear relevance, ambiguous wording, and limited follow-up point to several implementation priorities: explaining the purpose and relevance of PROMs, streamlining assessment content, tailoring administration and feedback to patient preferences, and creating actionable pathways for concerning results.

Participants strongly emphasized the necessity of acting on PROM results, echoing growing evidence that their clinical value hinges on visible translation into patient care [25]. Administering PROMs to patients establishes expectations that clinicians will use this information during consultations [20]. Failure to address patient responses during visits can result in disappointment and decreased likelihood of future PROM completion [19, 21]. When PROM results are not visibly translated into patient care, patient motivation to complete them declines, leading to increased missing data and compromised data quality, which limits their utility for clinical decision-making [26]. Visible action on PROMs—such as clinicians referencing PROM scores during visits (e.g., “I noticed your sleep score increased; let’s explore possible causes”) or generating patient-facing summaries—demonstrates that patient input is valued, thereby strengthening trust and improving data quality for both clinical and research purposes. Additionally, evidence from large pragmatic trials indicates that routine electronic symptom monitoring, when linked to actionable clinical responses, enhances patient-centered outcomes and healthcare utilization, emphasizing the importance of connecting clinical action to PROMs collected during visits [27].

Several participants perceived certain PROMs, particularly those addressing mental health, sexual health, or pain unrelated to their urology visit, as irrelevant to their clinical experience and expressed uncertainty or discomfort about the purpose of including such questions. Retzer et al. have emphasized the importance of measuring PRO concepts that hold relevance and utility to the target patient population [28]. However, static PROMs may include questions that are not applicable to specific sub-groups of patients within the target population [29]. Indeed, patients are skeptical of the relevance of PROMs due to healthcare professionals’ insufficient explanation of their purpose [30]. Without this background, even well-validated PROMs may be viewed as burdensome or irrelevant, thereby reducing completion rates and compromising data quality [31, 32]. Ensuring that patients understand the clinical rationale for including “non-urologic” questions is, therefore, a critical component of successful PROM implementation [33]. Our findings suggest that this issue was not simply resistance to a broader assessment approach, as participants endorsed a more holistic view of care, yet still had uncertainty about the relevance of some questions in the context of their LUTS/BPH care. This highlights that acceptance of broader PROM domains may depend not only on what is asked, but also on whether patients understand why these questions are being included and how identified concerns would be addressed. This finding directly supports the implementation strategies proposed in our study, including patient education, preference-sensitive workflows, and visible action on PROM results.

Participants expressed diverse preferences for communicating PROM results and managing health issues outside their physician’s practice. Participants expressed a desire to be consulted when non-urologic concerns identified by PROMs, such as stress, sleep problems, or depression, and to indicate whether they wanted their urologist to address them or refer them to another provider. These perspectives underscore the importance of designing PROM workflows that respect patient choice and autonomy. Embedding a patient-preference workflow within the EHR could operationalize this approach by enabling patients to opt out of non-urology-specific PROMs, select their preferred feedback delivery method, and specify which providers may access their results. Prior research demonstrates that tailoring PROM collection to patient preferences enhances both uptake and data quality [34]. This approach aligns with implementation science principles, which emphasize adapting interventions to patient needs and engaging patients as active stakeholders in the implementation process to enhance acceptability, uptake, and sustainability [35].

### Strengths and limitations

This study has several important strengths. It offers an in-depth, interpretive description of patient perspectives on participating in an early-stage PROM integration initiative. We recruited patients with diverse clinical symptom profiles and representation across both medical and surgical treatment pathways, enabling the capture of varied experiences across the LUTS/BPH care continuum. Additionally, patient feedback was translated into actionable implementation strategies suitable for adoption in clinical practice. Nonetheless, several limitations should be acknowledged. The generalizability of these findings is constrained by the demographic homogeneity of the sample, as all participants were White, non-Hispanic individuals recruited from a single health system. Preferences regarding PROM administration, patient portal communication, willingness to discuss sexual and mental health, and perceptions of the relevance of non-urologic domains may vary across racial, ethnic, linguistic, cultural, socioeconomic, and health-literacy groups [36–38]. Such differences are particularly relevant when PROM batteries include sensitive domains or depend on digital completion and electronic communication. Consequently, the patient-informed strategies identified in this study should not be presumed universally applicable. Future research should assess these strategies in more diverse patient populations, across both community and academic practice settings, and among individuals with varying levels of digital access, health literacy, and comfort discussing sensitive health topics [39].

As with other qualitative studies, selection bias is possible: patients who agreed to participate may have been more engaged in their care and more positively disposed toward PROMs than those who declined. It is important to note that the researchers’ own experiences and preconceptions may have influenced their interpretation of the data. To mitigate potential bias, two researchers with diverse disciplinary backgrounds collaboratively interpreted the data [40]. It is important to note that participants completed a comprehensive, LURN-informed assessment battery rather than the minimal PROM set typically used in routine urologic care. This expanded battery was selected to facilitate phenotyping and to capture domains relevant to LUTS presentation and severity. Our findings should not be interpreted as an endorsement of all these measures for routine clinical practice. Instead, the results identify which aspects of a broader assessment patients perceive as acceptable, confusing, burdensome, or clinically meaningful, and may inform the future development of a more streamlined, feasible, and patient-centered PROM workflow for routine LUTS/BPH care.

### Future directions

Our findings provide patient-informed guidance on integrating PROMs into clinical practice in ways that are more acceptable and meaningful to patients. However, successful integration of PROMs into routine care also requires consideration of clinician workflow, administrative feasibility, EHR infrastructure, and resource availability. Future implementation research should build on the patient-informed priorities identified in this study by incorporating clinician, administrator, and health-system perspectives. Such research could examine efficient review of PROM results during clinical visits, the definition of clinically actionable thresholds or referral pathways, and the integration of PROM data into EHR systems without imposing unnecessary burden on patients or care teams.

In LUTS/BPH care, future research should determine which PROM domains offer the most actionable value for both patients and clinicians. While the AUA-SI remains the traditional standard, it does not assess key symptom domains such as urinary incontinence and urinary pain. The LURN SI-29 addresses some of these gaps by capturing a broader spectrum of LUTS experiences. Building on this foundation, subsequent studies should further refine the PROM battery to preserve clinically meaningful and actionable domains, including relevant non-urologic areas such as sleep disturbance, anxiety, physical function, and pain, which may serve as modifiable predictors of LUTS severity. However, future research should not be limited to identifying which domains are statistically associated with LUTS/BPH outcomes but should also determine which domains clinicians can act on, which results patients want discussed, and which assessment components can be integrated into routine care without imposing an excessive burden.

Although this study focused on LUTS/BPH care, several findings may be transferable to PROM implementation in other medical specialties. Across clinical contexts, patients may be less inclined to complete PROMs if their purpose is unclear, if questionnaires are perceived as burdensome or insufficiently tailored, or if results are not reviewed or acted upon by clinicians. Consequently, the patient-centered strategies identified in this study—including clear communication, preference-sensitive workflows, flexible administration, tailored feedback, and actionable clinical responses—should be evaluated across diverse clinical settings.

## Conclusions

Patients with BPH recognize the potential benefits of PROM collection for monitoring and tracking symptoms and for improving communication. However, barriers identified—such as insufficient explanation of PROM purpose, perceived irrelevance of specific items, and lack of visible follow-up—may affect patient engagement and PROM completion. A multilevel implementation strategy is necessary to overcome these obstacles. Clinical practices can improve PROM integration by clearly explaining the purpose of PROMs, streamlining assessment content, tailoring administration to patient preferences, ensuring that results are reviewed during visits, and creating actionable pathways for concerns identified through PROMs. Future research should examine how these patient-informed strategies can be operationalized within clinical practice and integrated with clinician, administrator, and EHR perspectives to support sustainable PROM use in routine practice.

## Supplementary Information

Below is the link to the electronic supplementary material.

Supplementary Material 1

Supplementary Material 2

## Data Availability

The datasets used and/or analyzed during the current study are available from the corresponding author on reasonable request.

## Abbreviations

AUA-SI: American Urological Association Symptom Index
BPH: Benign Prostatic Hyperplasia
BMI: Body Mass Index
CMS: Centers for Medicare & Medicaid Services
COREQ: Consolidated Criteria for Reporting Qualitative Research
CPAP: Continuous Positive Airway Pressure
EHR: Electronic Health Record
HIPAA: Health Insurance Portability and Accountability Act
LURN: Symptoms of Lower Urinary Tract Dysfunction Research Network
LURN SI-10: Lower Urinary Tract Dysfunction Research Network Symptom Index-10
LURN SI-29: Lower Urinary Tract Dysfunction Research Network Symptom Index-29
LUTS: Lower Urinary Tract Symptoms
LUTS/BPH: Lower Urinary Tract Symptoms Attributed to Benign Prostatic Hyperplasia
OCF: Oncology Care First
PROM: Patient-Reported Outcome Measure
PROMIS: Patient-Reported Outcomes Measurement Information System
QoL: Quality of Life
REDCap: Research Electronic Data Capture
