# Supplementary material for: Gaps in patient-reported outcome measures integration into the management of lower urinary tract symptoms attributed to benign prostatic hyperplasia (LUTS/BPH): qualitative insights for implementation strategies
Source: J Patient Rep Outcomes. 2026 Jun 12;10:152. doi: 10.1186/s41687-026-01114-0 (PMC13486413; doi:10.1186/s41687-026-01114-0)
Supplement: Supplementary file 1 [file 41687_2026_1114_MOESM2_ESM.pdf]

Supplemental Material S2

Gaps in Patient-Reported Outcome Measures Integration into the Management of Lower Urinary Tract Symptoms Attributed to Benign Prostatic Hyperplasia (LUTS/ BPH): Qualitative Insights for Implementation Strategies

List of the patient reported outcome measures PROMs administered
1. LURN SYMPTOM INDEX-29 (LURN SI-29) [1]
2. Patient-Reported Outcomes Measurement Information System (PROMIS–29) Profile v2.1 [2]
3. The American Urological Association Symptom Index (AUA-SI) [3]
4. Patient Global Impression of Improvement (PGI-I) [4]
5. National Cancer Institute Quick Food Scan [5]
6. 3-Day Voiding Diary [6]
7. PROMIS® Squale v1.0 – Gastrointestinal Constipation 9a [7]
8. PROMIS® Scale v1.0 – Gastrointestinal Diarrhea 6a [7]
9. Sexual Health Inventory for Men (SHIM) [8]
10. Male Sexual Health Questionnaire-Ejaculatory Dysfunction (MSHQ-Ed) [9]
11. STOP-BANG questionnaire (SBQ) [10]
12. PROMIS® Item Bank v2.0 – Cognitive Function- Short Form 4a [11]
**13.** Patient Feedback Survey (optional)* [12]
*Adapted from Atkinson et. al. [12]

Supplemental Material S2

REFERENCE:

1. Cella D, Smith AR, Griffith JW, et al (2019) A New Outcome Measure for LUTS: Symptoms of Lower Urinary Tract Dysfunction Research Network Symptom Index-29 (LURN SI-29) Questionnaire. Neurourol Urodyn 38:1751. https://doi.org/10.1002/NAU.24067

2. Cella D, Riley W, Stone A, et al (2010) The Patient-Reported Outcomes Measurement Information System (PROMIS) developed and tested its first wave of adult self-reported health outcome item banks: 2005–2008. J Clin Epidemiol 63:1179–1194. https://doi.org/10.1016/j.jclinepi.2010.04.011

3. Barry MJ, Fowler FJ, O'Leary MP, et al (1992) The American Urological Association Symptom Index for Benign Prostatic Hyperplasia. Journal of Urology 148:1549–1557. https://doi.org/10.1016/S0022-5347(17)36966-5

4. Guy William, Branch. NI of MH (U. S). PR, Program. ECDE (1976) ECDEU assessment manual for psychopharmacology, Rev. U. S. Dept. of Health, Education, and Welfare, Public Health Service, Alcohol, Drug Abuse, and Mental Health Administration, National Institute of Mental Health, Psychopharmacology Research Branch, Division of Extramural Research Programs, Rockville, Md

5. Division of Cancer Control and Population Sciences (DCCPS) at The National Cancer Institute (NCI) operating under the National Institutes of Health (NIH) Eating at America ' s Table Study: Quick food Scan. In: Downloadable Dietary Assessment Screeners, Methodological Information, and Analytic Files

6. Bright E, Cotterill N, Drake M, Abrams P (2014) Developing and Validating the International Consultation on Incontinence Questionnaire Bladder Diary. Eur Urol 66:294–300. https://doi.org/10.1016/j.eururo.2014.02.057

7. Spiegel BMR, Hays RD, Bolus R, et al (2014) Development of the NIH Patient-Reported Outcomes Measurement Information System (PROMIS) Gastrointestinal Symptom Scales. American Journal of Gastroenterology 109:1804–1814. https://doi.org/10.1038/ajg.2014.237

8. Rosen R, Cappelleri J, Smith M, et al (1999) Development and evaluation of an abridged, 5-item version of the International Index of Erectile Function (IIEF-5) as a diagnostic tool for erectile dysfunction. Int J Impot Res 11:319–326. https://doi.org/10.1038/sj.ijir.3900472

9. Rosen RC, Catania JA, Althof SE, et al (2007) Development and Validation of Four-Item Version of Male Sexual Health Questionnaire to Assess Ejaculatory Dysfunction. Urology 69:805–809. https://doi.org/10.1016/j.urology.2007.02.036

10. Chung F, Yegneswaran B, Liao P, et al (2008) STOP Questionnaire. Anesthesiology 108:812–821. https://doi.org/10.1097/ALN.0b013e31816d83e4

11. Lai J-S, Wagner LI, Jacobsen PB, Cella D (2014) Self-reported cognitive concerns and abilities: two sides of one coin? Psychooncology 23:1133–1141. https://doi.org/10.1002/pon.3522

12. Atkinson TM, Schwartz CE, Goldstein L, et al (2019) Perceptions of Response Burden Associated with Completion of Patient-Reported Outcome Assessments in Oncology. Value in Health 22:225–230. https://doi.org/10.1016/j.jval.2018.07.875

LURN SYMPTOM INDEX-29 (LURN SI-29)

Instruction: This questionnaire asks you about different urinary symptoms. Please read each question carefully, and then circle the response that best describes your symptoms.

Section A

	Never	A few times	About half the time	Most of the time	Every time
**1.** In the past 7 days, how often did you completely lose control of your bladder?	0	1	2	3	4
**2.** In the past 7 days, how often did you leak urine or wet a pad after feeling a sudden need to urinate?	0	1	2	3	4
**3.** In the past 7 days, how often did you leak urine or wet a pad while laughing, sneezing, or coughing?	0	1	2	3	4
**4.** In the past 7 days, how often did you leak urine or wet a pad when doing physical activities, such as exercising or lifting a heavy object?	0	1	2	3	4
**5.** In the past 7 days, how often did walking at your usual speed cause you to leak urine or wet a pad?	0	1	2	3	4

	Never	A few nights	About half the nights	Most nights	Every night
**6.** In the past 7 days, how often did you leak urine during the night, including wetting a pad or the bed?	0	1	2	3	4

Section B

	Never	A few times	About half the time	Most of the time	Every time
**7.** In the past 7 days, how often did you have pain or discomfort in your bladder while it was filling?	0	1	2	3	4
**8.** In the past 7 days, how often did you have pain or discomfort in your bladder when it was full?	0	1	2	3	4
**9.** In the past 7 days, how often did you have pain or discomfort while urinating?	0	1	2	3	4
**10.** In the past 7 days, how often did you have pain or discomfort right after you had finished urinating?	0	1	2	3	4

Copyright © 2019

v1.1
Version Date 04/16/2021

Cella D, Smith AR, Griffith JW, et al. A new outcome measure for LUTS: Symptoms of Lower Urinary Tract Dysfunction Research Network Symptom Index-29 (LURN SI-29) questionnaire. *Neurourology and Urodynamics.* 2019;1-9. https://doi.org/10.1002/nau.24067

Section C

	Never	A few times	About half the time	Most of the time	Every time
**11.** In the past 7 days, how often did you have to push when urinating?	0	1	2	3	4
**12**. In the past 7 days, how often did you have a delay before you started to urinate?	0	1	2	3	4
**13.** In the past 7 days, once you started urinating, how often did your urine flow stop and start again?	0	1	2	3	4
**14.** In the past 7 days, how often was your urine flow slow or weak?	0	1	2	3	4
**15.** In the past 7 days, how often did you have a trickle or dribble at the end of your urine flow?	0	1	2	3	4

Section D

	Never	A few times	About half the time	Most of the time	Every time
**16.** In the past 7 days, how often did you feel a sudden need to urinate?	0	1	2	3	4
**17.** In the past 7 days, how often did you have a sudden need to rush to urinate for fear of leaking urine?	0	1	2	3	4

	Not difficult	A little difficult	Somewhat difficult	Very difficult	Unable to wait
**18.** In the past 7 days, once you noticed the need to urinate, how difficult was it to wait more than a few minutes?	0	1	2	3	4

Section E

	None	1 time	2-3 times	More than 3 times
**19.** In the past 7 days, during a typical night, how many times did you wake up and urinate? **Circle number here --->**	0	1	2	3

	Never	A few nights	About half the nights	Most nights	Every night
**20.** In the past 7 days, how often did you wake up at least once during the night because you had to urinate?	0	1	2	3	4

Copyright © 2019

Cella D, Smith AR, Griffith JW, et al. A new outcome measure for LUTS: Symptoms of Lower Urinary Tract Dysfunction Research Network Symptom Index-29 (LURN SI-29) questionnaire. *Neurourology and Urodynamics.* 2019;1-9. https://doi.org/10.1002/nau.24067

Section F

**21.** In the past 7 days, during waking hours, how many times did you typically urinate? Circle number here --->	**3 or fewer times a day** 0	**4-7 times a day** 1	**8-10 times a day** 2	**11 or more times a day** 3	
**22.** In the past 7 days, during a typical day, how much time typically passed between urinations? Circle number here --->	**More than 6 hours** 0	**5-6 hours** 1	**3-4 hours** 2	**1-2 hours** 3	**Less than 1 hour** 4
**23.** In the past 7 days, how would you describe your typical urge to urinate when you woke up during the night? Circle number here --->	**No urge** 0	**Mild urge** 1	**Moderate urge** 2	**Strong urge** 3	
**24.** In the past 7 days, did you have a constant need to urinate that did not go away? Circle number here --->	**Yes** 1	**No** 0			

	**Never**	**A few times**	**About half the time**	**Most of the time**	**Every time**
**25.** In the past 7 days, how often did you feel that your bladder was not completely empty after urination?	0	1	2	3	4
**26.** In the past 7 days, how often did you dribble urine just after zipping your pants or pulling up your underwear?	0	1	2	3	4
**FOR WOMEN ONLY:** **27a.** In the past 7 days, how often did you have spraying or change in direction of your urine stream?	0	1	2	3	4
**FOR MEN ONLY:** **27b**. In the past 7 days, how often did you have splitting or spraying of your urine stream?	0	1	2	3	4

**28.** In the past 7 days, how bothered were you by urinary symptoms? Circle number here --->	**Not at all bothered** 0	**Somwhat bothered** 1	**Very bothered** 2	**Extremely bothered** 3

Copyright © 2019

Cella D, Smith AR, Griffith JW, et al. A new outcome measure for LUTS: Symptoms of Lower Urinary Tract Dysfunction Research Network Symptom Index-29 (LURN SI-29) questionnaire. *Neurourology and Urodynamics.* 2019;1-9. https://doi.org/10.1002/nau.24067

PROMIS–29 Profile v2.1

Please respond to each question or statement by marking one box per row.

	**Physical Function**	**Without any difficulty**	**With a little difficulty**	**With some difficulty**	**With much difficulty**	**Unable to do**
PFA11	Are you able to do chores such as vacuuming or yard work?..........................	☐ 5	☐ 4	☐ 3	☐ 2	☐ 1
PFA21	Are you able to go up and down stairs at a normal pace?..	☐ 5	☐ 4	☐ 3	☐ 2	☐ 1
PFA23	Are you able to go for a walk of at least 15 minutes?...	☐ 5	☐ 4	☐ 3	☐ 2	☐ 1
PFA53	Are you able to run errands and shop?......	☐ 5	☐ 4	☐ 3	☐ 2	☐ 1

	**Anxiety**<br>**In the past 7 days…**	**Never**	**Rarely**	**Sometimes**	**Often**	**Always**
EDANX01	I felt fearful..	☐ 1	☐ 2	☐ 3	☐ 4	☐ 5
EDANX40	I found it hard to focus on anything other than my anxiety	☐ 1	☐ 2	☐ 3	☐ 4	☐ 5
EDANX41	My worries overwhelmed me...................	☐ 1	☐ 2	☐ 3	☐ 4	☐ 5
EDANX53	I felt uneasy	☐ 1	☐ 2	☐ 3	☐ 4	☐ 5

	**Depression**<br>**In the past 7 days...**	**Never**	**Rarely**	**Sometimes**	**Often**	**Always**
EDDEP04	I felt worthless	☐ 1	☐ 2	☐ 3	☐ 4	☐ 5
EDDEP06	I felt helpless..	☐ 1	☐ 2	☐ 3	☐ 4	☐ 5
EDDEP29	I felt depressed..	☐ 1	☐ 2	☐ 3	☐ 4	☐ 5
EDDEP41	I felt hopeless..	☐ 1	☐ 2	☐ 3	☐ 4	☐ 5

	**Fatigue**<br>**During the past 7 days…**	**Not at all**	**A little bit**	**Somewhat**	**Quite a bit**	**Very much**
HI7	I feel fatigued	☐ 1	☐ 2	☐ 3	☐ 4	☐ 5
AN3	I have trouble starting things because I am tired..	☐ 1	☐ 2	☐ 3	☐ 4	☐ 5

© 2008-2018 PROMIS Health Organization (PHO)

PROMIS–29 Profile v2.1

Fatigue

	In the past 7 days…	Not at all	A little bit	Somewhat	Quite a bit	Very much
FATEXP41	How run-down did you feel on average? ...	☐ 1	☐ 2	☐ 3	☐ 4	☐ 5
FATEXP40	How fatigued were you on average?	☐ 1	☐ 2	☐ 3	☐ 4	☐ 5

Sleep Disturbance

	In the past 7 days…	Very poor	Poor	Fair	Good	Very good
Sleep109	My sleep quality was.................................	☐ 5	☐ 4	☐ 3	☐ 2	☐ 1

	In the past 7 days…	Not at all	A little bit	Somewhat	Quite a bit	Very much
Sleep116	My sleep was refreshing............................	☐ 5	☐ 4	☐ 3	☐ 2	☐ 1
Sleep20	I had a problem with my sleep	☐ 1	☐ 2	☐ 3	☐ 4	☐ 5
Sleep44	I had difficulty falling asleep	☐ 1	☐ 2	☐ 3	☐ 4	☐ 5

Ability to Participate in Social Roles and Activities

		Never	Rarely	Sometimes	Usually	Always
SRPPER11_CaPS	I have trouble doing all of my regular leisure activities with others....................	☐ 5	☐ 4	☐ 3	☐ 2	☐ 1
SRPPER18_CaPS	I have trouble doing all of the family activities that I want to do	☐ 5	☐ 4	☐ 3	☐ 2	☐ 1
SRPPER23_CaPS	I have trouble doing all of my usual work (include work at home)	☐ 5	☐ 4	☐ 3	☐ 2	☐ 1
SRPPER46_CaPS	I have trouble doing all of the activities with friends that I want to do	☐ 5	☐ 4	☐ 3	☐ 2	☐ 1

Pain Interference

	In the past 7 days…	Not at all	A little bit	Somewhat	Quite a bit	Very much
PAININ9	How much did pain interfere with your day to day activities?.................................	☐ 1	☐ 2	☐ 3	☐ 4	☐ 5
PAININ22	How much did pain interfere with work around the home?	☐ 1	☐ 2	☐ 3	☐ 4	☐ 5
PAININ31	How much did pain interfere with your ability to participate in social activities? .	☐ 1	☐ 2	☐ 3	☐ 4	☐ 5
PAININ34	How much did pain interfere with your household chores?	☐ 1	☐ 2	☐ 3	☐ 4	☐ 5

© 2008-2018 PROMIS Health Organization (PHO)

PROMIS–29 Profile v2.1

Pain Intensity
In the past 7 days…

		0	1	2	3	4	5	6	7	8	9	10
Global07	How would you rate your pain on average?...	☐ 0 **No pain**	☐ 1	☐ 2	☐ 3	☐ 4	☐ 5	☐ 6	☐ 7	☐ 8	☐ 9	☐ 10 **Worst pain imaginable**

© 2008-2018 PROMIS Health Organization (PHO)

Urology Care FOUNDATION™
The Official Foundation of the American Urological Association

AMERICAN UROLOGICAL ASSOCIATION (AUA) SYMPTOM SCORE

NAME: ______________________________________

Do you have any problems when you urinate? We recommend that you talk with a health care provider if your total score on the first seven questions is 8 or greater or if you are bothered at all.

Have you noticed any of the following when you have gone to the bathroom to urinate over the past month? Circle the correct answer for you and write your score in the right-hand column.

	Not at all	Less than 1 time in 5	Less than half the time	About half the time	More than half the time	Almost always	Your Score
**Incomplete emptying** – It does not feel like I empty my bladder all the way.	0	1	2	3	4	5	
**Frequency** – I have to go again less than two hours after I finish urinating.	0	1	2	3	4	5	
**Intermittency** – I stop and start again several times when I urinate.	0	1	2	3	4	5	
**Urgency** – It is hard to wait when I have to urinate.	0	1	2	3	4	5	
**Weak stream** – I have a weak urinary stream.	0	1	2	3	4	5	
**Straining** – I have to push or strain to begin urination.	0	1	2	3	4	5	
	**None**	**1 time**	**2 times**	**3 times**	**4 times**	**5 times or more**	**Your Score**
**Nocturia** – I get up to urinate after I go to bed until the time I get up in the morning.	0	1	2	3	4	5	

Total AUA Symptom Score
Total score: 0-7 mild symptoms; 8-19 moderate symptoms; 20-35 severe symptoms

Quality of life due to urinary symptoms							
If you were to spend the rest of your life with your urinary condition the way it is now, how would you feel about that?	Delighted	Pleased	Mostly satisfied	Mixed: about equally satisfied and dissatisfied	Mostly dissatisfied	Unhappy	Terrible

Study ID #: ________________ **Date:** ________________
MM/DD/YYYY

Patient Global Impression of Improvement (PGI-I)

**Medical Management Study Visit**	Week 4	Week 8	Week 12 (clinic)	Week 16	Week 20	Week 24 (clinic)	
	☐	☐	☐	☐	☐	☐	
**Surgical Management Study Visit**	Week 1	Week 2	Week 4 (clinic)	Week 6	Week 8	Week 12 (clinic)	Week 24 (clinic)
	☐	☐	☐	☐	☐	☐	☐

<u>Instructions:</u> Check the one number that best describes how your bladder condition is now, compared with how it was before you began your Benign Prostatic Hyperplasia (BPH) treatment.

☐ 1 - Very much better

☐ 2 - Much better

☐ 3 - A little better

☐ 4 - No change

☐ 5 - A little worse

☐ 6 - Much worse

☐ 7 - Very much worse

Staff Initials: ________ Date Data Entered: ____________

ID # Place Label Here

NATIONAL CANCER INSTITUTE
QUICK FOOD SCAN

1. Think about your eating habits over the past 12 months. About how often did you eat or drink each of the following foods? Remember breakfast, lunch, dinner, snacks, and eating out. Blacken in only one bubble for each food.

TYPE OF FOOD	Never	Less than Once Per Month	1-3 Times Per Month	1-2 Times Per Week	3-4 Times Per Week	5-6 Times Per Week	1 Time Per Day	2 or More Times Per Day
Cold cereal	○	○	○	○	○	○	○	○
Skim milk, on cereal or to drink	○	○	○	○	○	○	○	○
Eggs, fried or scrambled in margarine, butter, or oil	○	○	○	○	○	○	○	○
Sausage or bacon, regular-fat	○	○	○	○	○	○	○	○
Margarine or butter on bread, rolls, pancakes	○	○	○	○	○	○	○	○
Orange juice or grapefruit juice	○	○	○	○	○	○	○	○
Fruit (not juices)	○	○	○	○	○	○	○	○
Beef or pork hot dogs, regular-fat	○	○	○	○	○	○	○	○
Cheese or cheese spread, regular-fat	○	○	○	○	○	○	○	○
French fries, home fries, or hash brown potatoes	○	○	○	○	○	○	○	○
Margarine or butter on vegetables, including potatoes	○	○	○	○	○	○	○	○
Mayonnaise, regular-fat	○	○	○	○	○	○	○	○
Salad dressings, regular-fat	○	○	○	○	○	○	○	○
Rice	○	○	○	○	○	○	○	○
Margarine, butter, or oil on rice or pasta	○	○	○	○	○	○	○	○

2. Over the past 12 months, when you prepared foods with margarine or ate margarine, how often did you use a reduced-fat margarine?

○ DIDN T USE MARGARINE ○ Almost never ○ About 1/4 of the time ○ About 1/2 of the time ○ About 3/4 of the time ○ Almost always or always

3. Overall, when you think about the foods you ate over the past 12 months, would you say your diet was high, medium, or low in fat?

○ High ○ Medium ○ Low

Study ID #: ________________

3-Day Voiding Diary

Choose a 3-day period when it is convenient for you to measure and record the following:
(Please begin the diary at midnight on day 1.)

1. The time (including AM or PM) for each entry
2. Your awakening and bedtimes during that 3-day period
3. Drinks: The amount of fluid you drink and specific type of beverage
4. Bladder sensation: Enter the number that corresponds with how your bladder felt when you went to the toilet using these codes:
   a. 0 – If you had no sensation of needing to pass urine, but passed urine for "social reasons," for example, just before going out, or unsure where the next toilet is
   b. 1 – If you had a normal desire to pass urine and no urgency. "Urgency" is different from normal bladder feelings and is the sudden compelling desire to pass urine which is difficult to defer, or a sudden feeling that you need to pass urine and if you don't you will have an accident
   c. 2 – If you had urgency but it had passed away before you went to the toilet
   d. 3 – If you had urgency but managed to get to the toilet, still with urgency, but did not leak urine
   e. 4 – If you had urgency and could not get to the toilet in time so you leaked urine
5. Leak: Any unintended loss of urine not measurable in your container. Please indicate whether you leaked because of urgency, stress or an unknown/other reason.
   a. "Urge leakage" is loss of urine associated with urgency (defined in bladder sensation 1 above). It includes leaks when standing up from a seated position, on the way to the bathroom and with triggers such as the presence of running water.
   b. "Stress leakage" is loss of urine associated with activity such as bending over, sneezing, laughing, coughing, lifting, jogging, running, or walking briskly. If you are unsure if you leaked due to stress or urgency, please write "unknown/other."
6. Void Amount: The amount of fluid you void (urinate)
   a. Use an old measuring cup or mark off ounces on an old jar or can, and use that to measure (2 tablespoons = 1 ounce)
   b. A urinal may be provided to you by the Research Coordinator
   c. If you passed urine but couldn't measure it, please put a check in this column.
7. The activity you are doing when you leak or feel the need to void
8. Pads: If you change a pad, please put a check in this column

Below is a sample diary for your review.

Time	Drinks		Bladder Sensation	Leak (stress, urge, or unknown/other)	Void Amount (oz)	Activity when you leaked or had an urge	Pads
	Fluid Intake Amount (oz)	Type of Beverage					
6:20 a.m.			3		8 oz	awakening	
7:00 a.m.	8 oz	coffee					
9:20 a.m.			4	urge	6 oz	washing	✓
11:30 a.m.	8 oz	sparkling water					

Staff Initials: ________ Date Data Entered: ____________

Voiding Diary Day 1

Date: ___________________

Time	Drinks		Bladder Sensation (0-4)	Leak (stress, urge, or unknown/other)	Void Amount (oz) [✓ if unmeasured]	Activity when you leaked or had an urge	Pads (✓ if you changed a pad)
	Fluid Intake Amount (oz)	Type of Beverage					
**TOTAL**	**oz**				**oz**		

Staff Initials: _________ Date Data Entered: _____________

Voiding Diary Day 2

Date: ________________

Time	Drinks		Bladder Sensation (0-4)	Leak (stress, urge, or unknown/other)	Void Amount (oz) [✓ if unmeasured]	Activity when you leaked or had an urge	Pads (✓ if you changed a pad)
	Fluid Intake Amount (oz)	Type of Beverage					
**TOTAL**	**oz**				**oz**		

Staff Initials: ________ Date Data Entered: ____________

Voiding Diary Day 3

Date: ___________________

Time	Drinks		Bladder Sensation (0-4)	Leak (stress, urge, or unknown/other)	Void Amount (oz) [✓ if unmeasured]	Activity when you leaked or had an urge	Pads (✓ if you changed a pad)
	Fluid Intake Amount (oz)	Type of Beverage					
**TOTAL**	**oz**				**oz**		

Staff Initials: _________

Date Data Entered: _____________

Gastrointestinal Constipation

Please respond to each question or statement by marking one box.

In the past 7 days…

1<br>GISX63	How often did you pass very hard or lumpy stools?

- ☐ 1 Never → **If Never, go to #3**
- ☐ 2 One day
- ☐ 3 2-6 days
- ☐ 4 Once a day
- ☐ 5 More than once a day

2<br>GISX64	How much did hard or lumpy stools bother you?

- ☐ 1 Not at all
- ☐ 2 A little bit
- ☐ 3 Somewhat
- ☐ 4 Quite a bit
- ☐ 5 Very much

3<br>GISX65	How often did you strain while trying to have bowel movements?

- ☐ 1 Never → **If Never, go to #6**
- ☐ 2 Rarely
- ☐ 3 Sometimes
- ☐ 4 Often
- ☐ 5 Always

© 2010-2016 PROMIS Health Organization (PHO)

In the past 7 days…

4 GISX66	How much did you usually strain while trying to have a bowel movement?

- ☐ 1 Not at all
- ☐ 2 A little bit
- ☐ 3 Somewhat
- ☐ 4 Quite a bit
- ☐ 5 Very much

5 GISX67	How much did straining during bowel movements bother you?

- ☐ 1 Not at all
- ☐ 2 A little bit
- ☐ 3 Somewhat
- ☐ 4 Quite a bit
- ☐ 5 Very much

6 GISX68	How often did you feel pain in your rectum or anus while trying to have bowel movements?

- ☐ 1 Never → **If Never, go to #8**
- ☐ 2 Rarely
- ☐ 3 Sometimes
- ☐ 4 Often
- ☐ 5 Always

© 2010-2016 PROMIS Health Organization (PHO)

In the past 7 days…

7<br>GISX69	At its worst, how would you rate the pain in your rectum or anus during bowel movements?

- ☐ 1 Not bad at all
- ☐ 2 A little bad
- ☐ 3 Somewhat bad
- ☐ 4 Quite bad
- ☐ 5 Very bad

8<br>GISX72	How often after a bowel movement did you feel unfinished - that is, that you had not passed all your stool?

- ☐ 1 Never
- ☐ 2 Rarely
- ☐ 3 Sometimes
- ☐ 4 Often
- ☐ 5 Always

9<br>GISX74	How often did you use your finger or toilet paper to get out a stool?

- ☐ 1 Never
- ☐ 2 Rarely
- ☐ 3 Sometimes
- ☐ 4 Often
- ☐ 5 Always

© 2010-2016 PROMIS Health Organization (PHO)

Gastrointestinal Diarrhea

Please respond to each question or statement by marking one box.

In the past 7 days…

1 GISX38	How many days did you have loose or watery stools?

- ☐ 1 No days → **If No Days, go to #4**
- ☐ 2 1 day
- ☐ 3 2 days
- ☐ 4 3-5 days
- ☐ 5 6-7 days

2 GISX40	How much did having loose or watery stools interfere with your day-to-day activities?

- ☐ 1 Not at all
- ☐ 2 A little bit
- ☐ 3 Somewhat
- ☐ 4 Quite a bit
- ☐ 5 Very much

3 GISX41	How much did having loose or watery stools bother you?

- ☐ 1 Not at all
- ☐ 2 A little bit
- ☐ 3 Somewhat
- ☐ 4 Quite a bit
- ☐ 5 Very much

© 2010-2016 PROMIS Health Organization (PHO)

In the past 7 days…

4<br>GISX42	How often did you feel like you needed to empty your bowels right away or else you would have an accident?

- ☐ 1 Never → **If Never, you are finished.**
- ☐ 2 One time during the past 7 days
- ☐ 3 2-6 times during the past 7 days
- ☐ 4 Often once a day
- ☐ 5 More than once a day

5<br>GISX43	How much did feeling you needed to empty your bowels right away interfere with your day-to-day activities?

- ☐ 1 Not at all
- ☐ 2 A little bit
- ☐ 3 Somewhat
- ☐ 4 Quite a bit
- ☐ 5 Very much

6<br>GISX44	How much did feeling you needed to empty your bowels right away bother you?

- ☐ 1 Not at all
- ☐ 2 A little bit
- ☐ 3 Somewhat
- ☐ 4 Quite a bit
- ☐ 5 Very much

© 2010-2016 PROMIS Health Organization (PHO)

Study ID #: _________________ **Date:** _________________
MM/DD/YYYY

Sexual Health Inventory for Men (SHIM) Questionnaire

**Medical Management Study Visit**	Baseline (clinic)	Week 4	Week 8	Week 12 (clinic)	Week 16	Week 20	Week 24 (clinic)		
	☐	☐	☐	☐	☐	☐	☐		
**Surgical Management Study Visit**	Baseline (clinic)	Preop	Week 1	Week 2	Week 4 (clinic)	Week 6	Week 8	Week 12 (clinic)	Week 24 (clinic)
	☐	☐	☐	☐	☐	☐	☐	☐	☐

<u>Instructions:</u> For each question, note your answer by circling the number that approximates your belief.

Over the past six months. . .

1. How do you rate your confidence that you could get and keep an erection?
   a. 1 – Very low
   b. 2 – Low
   c. 3 – Moderate
   d. 4 – High
   e. 5 – Very high

2. When you had erections with sexual stimulation, how often were your erections hard enough for penetration (entering your partner)?
   a. 0 – No sexual activity
   b. 1 – Almost never or never
   c. 2 – A few times (much less than half the time)
   d. 3 – Sometimes (about half the time)
   e. 4 – Most times (much more than half the time)
   f. 5 – Almost always or always

Staff Initials: ________ Date Data Entered: ____________

3. During sexual intercourse, how often were you able to maintain your erection after you had penetrated (entered) your partner?
   a. 0 – Did not attempt intercourse
   b. 1 – Almost never or never
   c. 2 – A few times (much less than half the time)
   d. 3 – Sometimes (about half the time)
   e. 4 – Most times (much more than half the time)
   f. 5 – Almost always or always

4. During sexual intercourse, how difficult was it to maintain your erection to completion of intercourse?
   a. 0 – Did not attempt intercourse
   b. 1 – Extremely difficult
   c. 2 – Very difficult
   d. 3 – Difficult
   e. 4 – Slightly difficult
   f. 5 – Not difficult

5. When you attempted sexual intercourse, how often was it satisfactory for you?
   a. 0 – Did not attempt intercourse
   b. 1 – Almost never or never
   c. 2 – A few times (much less than half the time)
   d. 3 – Sometimes (about half the time)
   e. 4 – Most times (much more than half the time)
   f. 5 – Almost always or always

Adapted from: Rosen RC, Cappelleri JC, Smith MD, Lipsky J, Peña BM. Development and evaluation of an abridged, 5-item version of the International Index of Erectile Dysfunction (IIEF-5) as a diagnostic tool for erectile dysfunction. Int J Impot Res. 1999;11:319-326.

Staff Initials: ________ Date Data Entered: ____________

Study ID #: ________________ **Date:** ________________
MM/DD/YYYY

Male Sexual Health Questionnaire-Ejaculatory Dysfunction (MSHQ-EjD)

**Medical Management Study Visit**	Baseline (clinic)	Week 4	Week 8	Week 12 (clinic)	Week 16	Week 20	Week 24 (clinic)		
	☐	☐	☐	☐	☐	☐	☐		
**Surgical Management Study Visit**	Baseline (clinic)	Preop	Week 1	Week 2	Week 4 (clinic)	Week 6	Week 8	Week 12 (clinic)	Week 24 (clinic)
	☐	☐	☐	☐	☐	☐	☐	☐	☐

<u>Instructions:</u> For each question, note your answer by circling the number that approximates your belief.

In the past month. . .

1. How often have you been able to ejaculate or "cum" when having sexual activity?
   a. 1 – None of the time / could not ejaculate
   b. 2 – Less than half the time
   c. 3 – About half the time
   d. 4 – Most of the time
   e. 5 – All of the time

2. How would you rate the strength or force of your ejaculation?
   a. 0 – Could not ejaculate
   b. 1 – Very much less strong than it used to be
   c. 2 – Much less strong than it used to be
   d. 3 – Somewhat less strong than it used to be
   e. 4 – A little less strong than it used to be
   f. 5 – As strong as it always was

3. How would you rate the amount or volume of semen or fluid when you ejaculate?
   a. 0 – Could not ejaculate
   b. 1 – Very much less than it used to be
   c. 2 – Much less than it used to be
   d. 3 – Somewhat less than it used to be
   e. 4 – A little less than it used to be
   f. 5 – As much as it always was

Staff Initials: ________ Date Data Entered: ____________

4. If you have had any ejaculation difficulties or have been unable to ejaculate, have you been bothered by this?
   a. 0 – No problem with ejaculation
   b. 1 – Not at all bothered
   c. 2 – A little bothered
   d. 3 – Moderately bothered
   e. 4 – Very bothered
   f. 5 – Extremely bothered

Adapted from: UROLOGY 69: 805–809, 2007

Staff Initials: ________ Date Data Entered: ____________

STOP BANG Questionnaire

Height _____ inches/cm Weight _____ lb/kg
Age _____
Male/Female
BMI _____

Collar size of shirt: S, M, L, XL, or _____ inches/cm
Neck circumference* _____ cm

1. *S*noring
Do you *s*nore loudly (louder than talking or loud enough to be heard through closed doors)?
Yes No

2. *T*ired
Do you often feel *t*ired, fatigued, or sleepy during daytime?
Yes No

3. *O*bserved
Has anyone *o*bserved you stop breathing during your sleep?
Yes No

4. Blood *p*ressure
Do you have or are you being treated for high blood *p*ressure?
Yes No

5. *B*MI
*B*MI more than 35 kg/m^2?
Yes No

6. *A*ge
*A*ge over 50 yr old?
Yes No

7. *N*eck circumference
*N*eck circumference greater than 40 cm?
Yes No

8. *G*ender
*G*ender male?
Yes No

* Neck circumference is measured by staff
High risk of OSA: answering yes to three or more items
Low risk of OSA: answering yes to less than three items

Adapted from:
STOP Questionnaire
A Tool to Screen Patients for Obstructive Sleep Apnea
Frances Chung, F.R.C.P.C., Balaji Yegneswaran, M.B.B.S.,† Pu Liao, M.D.,‡ Sharon A. Chung, Ph.D.,§ Santhira Vairavanathan, M.B.B.S.,_ Sazzadul Islam, M.Sc.,_ Ali Khajehdehi, M.D.,† Colin M. Shapiro, F.R.C.P.C.#*
Anesthesiology 2008; 108:812–21 Copyright © 2008, the American Society of Anesthesiologists, Inc. Lippincott Williams & Wilkins, Inc.

Cognitive Function- Short Form 4a

Please respond to each question or statement by marking one box per row.

	**In the past 7 days...**	**Never**	**Rarely (Once)**	**Sometimes (Two or three times)**	**Often (About once a day)**	**Very often (Several times a day)**
PC2r	My thinking has been slow	☐ 5	☐ 4	☐ 3	☐ 2	☐ 1
PC35r	It has seemed like my brain was not working as well as usual	☐ 5	☐ 4	☐ 3	☐ 2	☐ 1
PC36r	I have had to work harder than usual to keep track of what I was doing	☐ 5	☐ 4	☐ 3	☐ 2	☐ 1
PC42r	I have had trouble shifting back and forth between different activities that require thinking	☐ 5	☐ 4	☐ 3	☐ 2	☐ 1

© 2010-2020 PROMIS Health Organization (PHO)

Study ID #: ________________ **Date:** ________________
MM/DD/YYYY

Patient Feedback Survey

1. How well did these questions relate to your actual concerns?

   0 1 2 3 4 5 6 7 8 9 10
   Not at all related Very related

2. How comfortable were you in answering questions?

   0 1 2 3 4 5 6 7 8 9 10
   Not at all comfortable Very comfortable

3. How well did these questions describe your health and well-being?

   0 1 2 3 4 5 6 7 8 9 10
   Not at all Very well

4. How did you feel about the length of time to complete these questionnaires?

   1 Much too long 2 A bit too long 3 Just right / no problem

5. What questions seemed unimportant or repetitive?

6. What additional information should we gather?

Staff Initials: ________ Date Data Entered: ____________
