# Supplementary material for: Gaps in patient-reported outcome measures integration into the management of lower urinary tract symptoms attributed to benign prostatic hyperplasia (LUTS/BPH): qualitative insights for implementation strategies
Source: J Patient Rep Outcomes. 2026 Jun 12;10:152. doi: 10.1186/s41687-026-01114-0 (PMC13486413; doi:10.1186/s41687-026-01114-0)
Supplement: Supplementary file 2 [file 41687_2026_1114_MOESM1_ESM.doc]

**Gaps in Patient-Reported Outcome Measures Integration into the Management of Lower Urinary Tract Symptoms Attributed to Benign Prostatic Hyperplasia (LUTS/BPH): Qualitative Insights for Implementation Strategies**

**Table S1:** Consolidated criteria for reporting qualitative studies (COREQ): 32-item checklist

| **No. Item** | **Guide questions/description** | **Section** |
| --- | --- | --- |
| **Domain 1: Research team and reﬂexivity** |  |  |
| ***Personal Characteristics*** |  |  |
| **1. Interviewer/facilitator** | Which author/s conducted the interview or focus group? | Methods—Procedures |
| **2. Credentials** | What were the researcher’s credentials? E.g. PhD, MD | Methods—Procedures |
| **3. Occupation** | What was their occupation at the time of the study? | Methods—Procedures |
| **4. Gender** | Was the researcher male or female? | Not reported |
| **5. Experience and training** | What experience or training did the researcher have? | Methods—Procedures |
| ***Relationship with participants*** |  |  |
| **6. Relationship established** | Was a relationship established prior to study commencement? | Methods—Procedures |
| **7. Participant knowledge of the interviewer** | What did the participants know about the researcher? e.g. personal goals, reasons for doing the research | Methods—Procedures |
| **8. Interviewer characteristics** | What characteristics were reported about the interviewer/facilitator? e.g. Bias, assumptions, reasons and interests in the research topic | Discussion—Strengths and Limitations |
| **Domain 2: study design** |  |  |
| ***Theoretical framework*** |  |  |
| **9. Methodological orientation and Theory** | What methodological orientation was stated to underpin the study? e.g. grounded theory, discourse analysis, ethnography, phenomenology, content analysis | Methods—Analysis |
| ***Participant selection*** |  |  |
| **10. Sampling** | How were participants selected? e.g. purposive, convenience, consecutive, snowball | Methods—Participants, Recruitment, and Setting |
| **11. Method of approach** | How were participants approached? e.g. face-to-face, telephone, mail, email | Methods—Participants, Recruitment, and Setting; Procedures |
| **12. Sample size** | How many participants were in the study? | Results (participant characteristics / opening paragraph); Methods—Procedures |
| **13. Non-participation** | How many people refused to participate or dropped out? Reasons? | Methods—Procedures |
| ***Setting*** |  |  |
| **14. Setting of data collection** | Where was the data collected? e.g. home, clinic, workplace | Methods—Procedures |
| **15. Presence of non-participants** | Was anyone else present besides the participants and researchers? | Methods—Procedures |
| **16. Description of sample** | What are the important characteristics of the sample? e.g. demographic data, date | Methods—Participants, Recruitment, and Setting; Results—Table 1 |
| ***Data collection*** |  |  |
| **17. Interview guide** | Were questions, prompts, guides provided by the authors? Was it pilot tested? | Methods—Procedures; Supplemental Material Table S2 |
| **18. Repeat interviews** | Were repeat interviews carried out? If yes, how many? | Not applicable |
| **19. Audio/visual recording** | Did the research use audio or visual recording to collect the data? | Methods—Procedures |
| **20. Field notes** | Were ﬁeld notes made during and/or after the interview or focus group? | Not applicable |
| **21. Duration** | What was the duration of the interviews or focus group? | Methods—Procedures |
| **22. Data saturation** | Was data saturation discussed? | Methods—Procedures |
| **23. Transcripts returned** | Where transcripts returned to participants for comment and/or correction? | No applicable |
| **Domain 3: analysis and ﬁndings** |  |  |
| ***Data analysis*** |  |  |
| **24. Number of data coders** | How many data coders coded the data? | Methods—Analysis |
| **25. Description of the coding tree** | Did authors provide a description of the coding tree? | Results (codebook referenced); Supplemental Material Table S3 |
| **26. Derivation of themes** | Were themes identiﬁed in advance or derived from the data? | Methods—Analysis |
| **27. Software** | What software, if applicable, was used to manage the data? | Methods—Analysis |
| **28. Participant checking** | Did participants provide feedback on the ﬁndings? | Not applicable |
| ***Reporting*** |  |  |
| **29. Quotations presented** | Were participant quotations presented to illustrate the themes/ﬁndings? Was each quotation identiﬁed? e.g. participant number | Methods—Procedures; Results (quotations by interview # and arm) |
| **30. Data and ﬁndings consistent** | Was there consistency between the data presented and the ﬁndings? | Results |
| **31. Clarity of major themes** | Were major themes clearly presented in the ﬁndings? | Results |
| **32. Clarity of minor themes** | Is there a description of diverse cases or discussion of minor themes? | Results (diverse preferences within themes, incl. Practical Considerations) |

Developed from: Tong A, Sainsbury P, Craig J. Consolidated criteria for reporting qualitative research (COREQ): a 32-item checklist for interviews and focus groups. *International Journal for Quality in Health Care*. 2007. Volume 19, Number 6: pp. 349 – 357

Table S2: Interview guide

**Q1: We would like your feedback on those questionnaires.**

- **What did you think of them?**
- **Which questionnaires do you think were the most related to your condition?**
- **Which questionnaires were the least useful?**
- **Is there anything you think is important that the questionnaires did not ask about? If so, what?**
- **How could these questionnaires be helpful for your health care?**

**Q2: For the questionnaires that asked about urinary symptoms, there were questions relating to pain during urination, leakage, split stream, and spraying while urinating.**

- **On a scale of 0 to 10 (with 0 being not important and 10 being very important), How would you rate these questions? Why did you give that rating?**

**Q3: During your urologist’s visit, was there anything that you wanted to bring up but didn’t? (Please note, you don't need to disclose specific details, we're interested in understanding any reasons or barriers you may have faced.)**

**Q4: In this study we are trying to better understand how to give patients feedback about questionnaires.**

- **How would you prefer the questionnaire results to be communicated to you? (Alt phrasing: How would you prefer to receive your questionnaire results?)**
- **How do you want the results from the questionnaires to be used in your medical care?**
- **If your urologist noticed symptoms that are not directly related to your prostate condition, such as trouble sleeping or feeling stressed, how would you like them to addressed?**

| **Table S3: Codebook** | |
| --- | --- |
| **Code** | **Description** |
| **Perceived benefits** | |
| 1. **Patient Engagement** | Belief that PROMs encourage patients to reflect on their symptoms, track progress, and actively participate in their care through better communication with providers. |
| 2. **Care Coordination** | Preference for sharing PROM results across the care team to improve collaboration, ensure all providers are informed, and streamline care delivery. |
| 3. **Clinically Meaningful Application** | PROMs perceived as valuable tools to support diagnosis, monitor disease progression, and guide timely adjustments to treatment plans. |
| **Questionnaires content** | |
| 1. **Perceptions of Questionnaires’ topics** *(****relevance, sensitive topics)*** | The perception of how closely PROMs align with a patient’s specific condition, while avoiding overly generic questions that may feel irrelevant or disconnected from their personal experience.  Questions perceived by patients as highly personal, intrusive, or uncomfortable, which may lead to skipped items, withheld information, or incomplete responses. |
| 2. **Perceptions of Response Options** | Preference for answer choices that capture greater nuance and flexibility, including opportunities to provide additional details or context beyond predetermined, fixed responses |
| 3. **Recall Periods** | Difficulties arising from fixed timeframes (e.g., “past 7 days”) when symptom patterns fluctuate, creating concerns that responses may not accurately reflect the patient’s usual or overall experience. |
| 4. **Perceived comprehensiveness** | The perception that PROMs battery administered fully capture the most important symptoms and experiences relevant to the patient. |
| 5. **Wording Clarity** | Need for clear, straightforward wording to avoid confusion and ensure consistent interpretation of items. |
| **Perceived Barriers** | |
| 1. **Physical Burden** | Physical discomfort experienced when completing PROMs, such as difficulty due to sleep disruption. |
| 2. **Overwhelming Quantity** | Patients' frustrations with excessive numbers of questionnaires administered |
| **Considerations for PROM Integration in routine clinical practice** | |
| 1. **Preferred Feedback Format** | Patients' preferred ways of receiving PROM results, such as via patient portals, in-person discussions, printed reports, or other formats. |
| 2. **Interpretation / Presentation of PROM Data** | Preferences for how PROM results are displayed (graphs vs. text) and explained |
| 3. **Actionable Recommendations** | Patients value receiving clear follow-up steps, advice, or treatment recommendations based on their PROM scores. |
| 4. **Whole-Person Symptom Recognition** | Support for holistic care that considers symptoms beyond the provider's specialty. |
| 5. **Patient Education about PROs** | Interest in understanding the relationship between symptoms measured in PROMs and their underlying condition |
